# Decentralization and Regionalization of Surgical Care: A Review of Evidence for the Optimal Distribution of Surgical Services in Low- and Middle-Income Countries

**DOI:** 10.15171/ijhpm.2019.43

**Published:** 2019-06-17

**Authors:** Katherine R. Iverson, Emma Svensson, Kristin Sonderman, Ernest J. Barthélemy, Isabelle Citron, Kerry A. Vaughan, Brittany L. Powell, John G. Meara, Mark G. Shrime

**Affiliations:** ^1^Program in Global Surgery and Social Change, Harvard Medical School, Boston, MA, USA.; ^2^General Surgery Department, University of California Davis Medical Center, Sacramento, CA, USA.; ^3^Lund University, Lund, Sweden.; ^4^Brigham and Women’s Hospital, Boston, MA, USA.; ^5^Icahn School of Medicine at Mount Sinai, New York City, NY, USA.; ^6^University of Pennsylvania, Philadelphia, PA, USA.; ^7^Stanford University School of Medicine, Stanford, CA, USA.; ^8^Department of Plastic and Oral Surgery, Boston Children’s Hospital, Boston, MA, USA.; ^9^Massachusetts Eye and Ear Infirmary, Boston, MA, USA.

**Keywords:** Global Surgery, Service Delivery, Regionalization, Decentralization, Centralization, Low- and Middle-Income Countries (LMICs)

## Abstract

**Background:** While recommendations for the optimal distribution of surgical services in high-income countries (HICs) exist, it is unclear how these translate to resource-limited settings. Given the significant shortage and maldistribution of surgical workforce and infrastructure in many low- and middle-income countries (LMICs), the optimal role of decentralization versus regionalization (centralization) of surgical care is unknown. The aim of this study is to review evidence around interventions aimed at redistributing surgical services in LMICs, to guide recommendations for the ideal organization of surgical services.

**Methods:** A narrative-based literature review was conducted to answer this question. Studies published in English between 1997 and 2017 in PubMed, describing interventions to decentralize or regionalize a surgical procedure in a LMIC, were included. Procedures were selected using the Disease Control Priorities’ (DCP3) Essential Surgery Package list. Intervention themes and outcomes were analyzed using a narrative, thematic synthesis approach. Primary outcomes included mortality, complications, and patient satisfaction. Secondary outcomes included input measures: workforce and infrastructure, and process measures: facility-based care, surgical volume, and referral rates.

**Results:** Thirty-five studies were included. Nine (33%) of the 27 studies describing decentralization showed an improvement in primary outcomes. The procedures associated with improved outcomes after decentralization included most obstetric, gynecological, and family planning services as well as some minor general surgery procedures. Out of 8 studies on regionalization (centralization), improved outcomes were shown for trauma care in one study and cataract extraction in one study.

**Conclusion:** Interventions aimed at decentralizing obstetric care to the district hospital and health center levels have resulted in mortality benefits in several countries. However, more evidence is needed to link service distribution to patient outcomes in order to provide recommendations for the optimal organization of other surgical procedures in LMICs. Considerations for the optimal distribution of surgical procedures should include the acuity of the condition for which the procedure is indicated, anticipated case volume, and required level of technical skills, resources, and infrastructure. These attributes should be considered within the context of each country.

## Introduction

Although there has been considerable progress in improving access to surgical, anesthetic, and obstetric care in many low- and middle-income countries (LMICs), little is known about the ideal distribution of these services. Considering the significant shortage and maldistribution of surgical workforce and infrastructure in these settings,^1,2^ the organization of surgical delivery must be optimized to ensure adequate access to safe and quality care. In 2015, the third volume of Disease Control Priorities (DCP3) published the Essential Surgery Package, consisting of 44 surgical procedures over 3 service delivery platforms that, if provided, could avert an estimated 1.5 million deaths a year.^3^ These procedures were chosen as the most essential and effective in restoring health and quality of life for the greatest number of people. Although providing much-needed guidance to the scale-up of surgical and obstetric care, evidence supporting the appropriate distribution of these services has not been well described. The role of decentralization versus regionalization of specific surgical services has yet to be established.

Decentralization of care is defined as the process of transferring authority, services, and decision-making power from central governance bodies to lower management levels.^4^ In healthcare, a decentralized system implies distributing health services closer to populations that may otherwise not have access to these services. Decentralization has been argued to increase accountability and improve effectiveness because of the spatial and temporal proximity to patients and the capability to adjust services according to local needs.^5^ Many LMICs have undergone health sector reforms in the last four decades, the majority of which have included some degree of decentralization. There has been increased momentum for this type of restructuring in recent years with universal health coverage, ensuring access to essential health services for all, becoming an international priority as evidenced by the United Nations’ Sustainable Development Goals. This reorganization has improved access to healthcare for rural populations by decreasing distance to health facilities and has been associated with improved outcomes including decreased mortality.^6-8^ However, much of this research has focused on primary healthcare or programs specific to tuberculosis or HIV care, with little to no evidence related to surgery.

Regionalization, also referred to as “centralization” in this paper, describes the process of managing resources (such as staffing and funding) from a central body, in order to concentrate expertise and resources in a few specialized institutions.^9,10^ In high-income countries (HICs), regionalization has been implemented mainly for health services with a high demand on technical and workforce skills, such as specialized oncology treatment or pediatric surgical care.^11-13^ Furthermore, creating high-volume centers has shown favorable outcomes for perinatal and trauma care.^14,15^ However, regionalization has also resulted in increased travel times^16^ and financial hardship for patients diminishing access to care for already vulnerable and poor populations as well as populations living in rural areas.^17,18^ The concentration of surgical services in urban areas in LMICs has been a byproduct of the overall shortage of surgical providers. There is often a default centralization of surgery due to the lack of personnel and infrastructure in communities outside of major cities. However, it is unclear whether these populations would be better served with redistribution of these services, or with enhancement of referral, transportation, and communication systems to increase access to surgical care in these major centers.

Considering the significant differences in settings and health system organization, recommendations around the role of decentralization or regionalization of surgical care in HICs cannot be directly translated to resource-limited locations.^11-13,17,19^ There is also a lack of evidence-based guidelines from LMICs for recommending the optimal distribution of surgical services. Therefore, the aim of this study is to review the current evidence around interventions seeking to decentralize or regionalize essential surgical services and analyze their impact on patient outcomes, safety, and quality of care. The goal is to provide recommendations for the optimal organization of surgical care in LMICs.

## Methods

### Search Strategy and Selection Criteria

A scoping, narrative review was performed in which PubMed was searched to identify articles describing interventions which sought to redistribute surgical services in LMICs and their related outcomes. Studies published in English between January 1997 and October 2017, describing (1) decentralization and/or regionalization of one or more (2) procedure(s) from the DCP3’s Essential Surgery Package list^3^ in (3) a low- or middle-income country were included. Dental procedures and normal deliveries were excluded from the search due to these procedures not requiring major surgery. All search terms are included in Supplementary file 1.

A total of 4011 records were identified. After screening titles and abstracts, 57 papers were included for full-text review. One paper was excluded because no full-text was available. One paper was excluded for containing no original data. Two papers were excluded for being conducted in HICs. Eighteen papers were excluded for not fitting the above inclusion criteria, leaving 35 papers after the full-text screening for inclusion in the review (Figure).^20^ Further quality assessment of the source data was not performed. This is due to the chosen analytical approach of a narrative analysis with extraction of primarily qualitative data.

**Figure:**
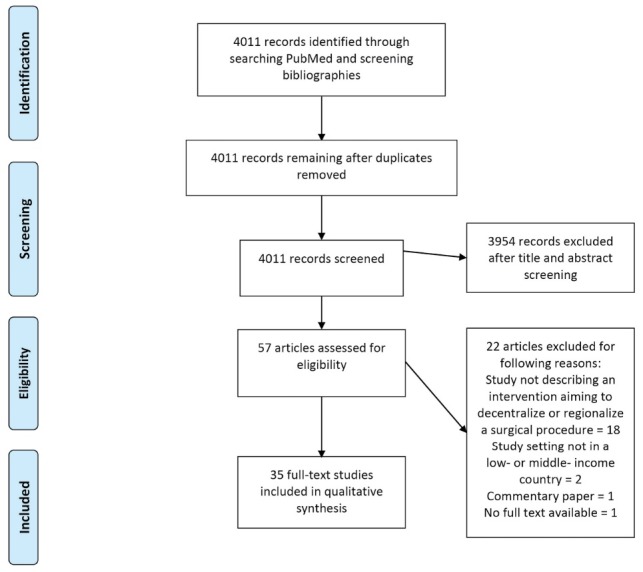
PRISMA Flow Chart Describing the Steps in Retrieving and Screening Records Including Search Strategy Results, Reasons for Exclusion, and Final Record Number.^20^

### Data Extraction

The following data were extracted from each study: country or countries for the intervention; category of the procedure as defined by the DCP3 Essential Surgery Package (Obstetric, gynecological, and family planning; General surgery; Injury; Congenital; Visual impairment, and Non-trauma orthopedic); the specific procedure or most common 3 procedures if multiple; whether the intervention aimed to decentralize or regionalize care; brief description of the intervention, study design, intervention category (workforce training, infrastructure/equipment/supplies, information system, community outreach, referral system/transportation, and service delivery organization); and main outcomes. Interventions aimed at increasing access to a surgical procedure in a rural area or at the health center or district hospital level were classified under “Decentralization.” Interventions directed at the regional hospital level or a centralized care center were included under “Regionalization.”

### Analysis

A narrative, thematic synthesis approach was used for analysis.^21,22^ The World Health Organization (WHO) health system building blocks were used to define the initial intervention themes.^23^ The WHO building blocks of “Financing” and “Leadership/Governance” were ultimately excluded due to the majority of included studies involving foreign investment and partnerships, and thus these 2 themes were less relevant. The 4 remaining themes from the WHO building blocks model included: workforce training, infrastructure/equipment/supplies, information system, and service delivery organization. Two additional areas were selected (community outreach and referral system/transportation) as they were commonly employed to execute the redistribution of care in these studies. The final intervention categories were included if they were common to at least 3 studies.

Eight separate outcome categories (increased workforce, increase in infrastructure, increased facility-based care, increased surgical volume or breadth, decreased referral rates, decreased mortality, decreased complications, and patient satisfaction) were extracted based on a Donabedian framework of input, process, and outcome results (Table 1).^24^ Outcome measures refer to the end result on the patient population and included: decreased mortality, decreased complications, and patient satisfaction. Primary outcomes included these patient outcomes, and secondary outcomes include the input and process measures as outlined in Table 1. Due to the focus on qualitative data extraction, further tests of data validity were not performed.

**Table 1:** Donabedian Framework Categorization of Outcomes Assessed in Each Study

**Input**	**Process**	**Outcome**
(1) Increased Workforce	(3) Increased facility-based care	(6) Decreased mortality
(2) Increase in Infrastructure	(4) Increased surgical volume or breadth	(7) Decreased complications
	(5) Decreased referral rates	(8) Patient satisfaction

## Results

### Decentralization

The majority of included studies (77%, n = 27) described interventions aiming to decentralize surgical care. Of these studies, the majority related to obstetric, gynecological, and family planning procedures (52%) and 26% specifically to delivery and obstetric care (Table 2).^25-51^ The most common decentralized procedures were obstetric care, cryotherapy for pre-cancerous cervical lesions, and male circumcision for HIV prevention. All but one intervention (96%) included workforce training of non-surgeon physicians or non-physician practitioners as a main component.^25-39,41-51^ The most common outputs from all decentralization studies were increased facility-based care and increased surgical volume or increased breadth of surgical procedures available (Table 2).

**Table 2:** Description of Studies Aimed at Decentralizing Surgical Services

**Decentralization**						
**Reference**	**Year**	**Country**	**Specific Procedure**	**Intervention Description**	**Outcome Description**	**Study Design**
**Obstetric, Gynecological, and Family Planning**						
Kestler et al^25^	2006	Guatemala	Post-abortion care: MVA	In-country professional group and government-led initiative to scale-up post-abortion care at 22/33 public district hospitals over 18-month period Categories: (A) (B) (C)	Process^3,4^ • *Increase in use of MVA for incomplete abortions from 38% to 68% • Increase in number of patients presenting during first trimester	Descriptive, pre- and post-data
Kiemtoré et al^26^	2017	Burkina Faso	Post-abortion care: MVA	In-country surgical society provided training and healthcare equipment in 45 rural, primary-level health facilities over one-year period Categories: (A) (B)	Process^3,4,5^ • *Increase in number of cases of incomplete abortion treated per year from 1812 to 2738 (*+51.1%) • Increase in # facilities offering post-abortion care • *Increase in MVA used to treat incomplete abortions (+97.6%) • *Decrease in referrals (-87.4%) • **Outcome**^7^ • *Decrease in complications of uterine perforation (-73.6%) and pelvic infection (-49.8%) after MVA	Descriptive longitudinal, pre- and post-data
Moon et al^27^	2012	Mozambique	Cryotherapy	International partnership to implement VIA screening and cryotherapy treatment in rural health facilities and clinics, year one of program Categories: (A) (B) (E)	Process^3,4^ • Increase in number of women undergoing cervical cancer screening by trained nurses • Increase in number of women treated by cryotherapy from 53% to 96%	Descriptive longitudinal
Ramogola-Masire et al^28^	2012	Botswana	Cryotherapy	International academic partnership implemented, community-based cervical cancer prevention program to train community clinic nurses and refer complicated cases over 23-month period Categories: (A)	Input^1^ • Local nurses trained to provide cryotherapy care • Process^3^ • 2175 women appropriately treated by community nurses • 264 pre-cancerous lesions treated	Descriptive cross-sectional
Kim et al^29^	2013	Indonesia	Cryotherapy	Government-led initiative, in collaboration with international partners, to disseminate VIA screening and cryotherapy services to 47 health centers over four-year period Categories: (A) (B) (C) (D)	Input^1^ • General practitioners, physicians, and midwives trained • Process^3,4^ • Increase in number of women screened per year from 4874 to 12 695 • % VIA treatable women seeking care increased from 63% to 83.1%	Retrospective cohort
Khozaim et al^30^	2014	Kenya	Cryotherapy	International partnership to expand cervical cancer screening and treatment to 4 public, regional health facilities over 26-month period Categories: (A) (B) (E)	Process^3,4^ • 6787 women screened • *31.5% of women requiring additional testing or procedures lost to follow-up*	Retrospective chart review
Poli et al^31^	2015	India	Cryotherapy	Community-based training program to train rural community health workers in VIA and cryotherapy over 7-year period Categories: (A) (D)	Input^1^ • Increased number of trained female health workers and medical officers • Process^3,4^ • 18 000 women screened, 312 women underwent cryotherapy • 108 referred to higher care, 49 underwent hysterectomy	Descriptive
Mekbib et al^32^	2003	Ethiopia	Normal delivery, Instrumental delivery, Caesarean section	International partnership with professional society to improve emergency obstetric availability in one district hospital and two health centers in one district of Ethiopia over 3 years Categories: (A) (B) (C) (D)	Input^1^ 7 general practitioners, 4 midwives, 5 health officers, 18 health assistants trained • Process^3,4,5^ • Increase in proportion of births at EmOC capable facilities by 39.7% • *Six-fold increase in c-section rates from 3.7% to 17.3%* • *Obstetric complications treated increased by 237%* • Outcome,^7^ • CFR for direct maternal deaths decreased from 7.2% in 1999 to 4.6% in 2001	Retrospective review, • prospective data collection, • pre- and post-data
Kayongo et al^33^	2006	Ethiopia, Rwanda, Tanzania	Normal delivery, Instrumental delivery, Caesarean section	International NGO aimed to build emergency obstetric capacity at 10 district hospitals over four years Categories: (A) (B) (C)	Process^3,4^ • Increased proportion of births in EmOC capable facilities • Tanzania: 14% to 18%; Rwanda: 8.9% to 8.8%; Ethiopia: 0.8% to 2.0% • Increased met need for EmOC services • Tanzania: 14% to 19%; Rwanda: 16% to 25%; Ethiopia: 2% to 4.5% • Increased c-section rate • Tanzania: 1.4% to 1.8%; Rwanda: 1.8% to 2.9%; Ethiopia: 0.1% to 9.4% • Outcome^6^ • CFR decreased by 30%-50% • Tanzania: 3.9% to 1.9% • Rwanda: 2.0% to 0.9% • Ethiopia: 7.8% to 5.2%	Descriptive longitudinal
Evans et al^34^	2009	India	Instrumental delivery, Caesarean section	Government and professional society led with international assistance, centralized c-section 16-week training program for medical officers in two states of rural India; outcomes evaluated 2 years later Categories: (A)	Input^1^ • 17 medical officers trained to provide EmOC at 15 different facilities • *8/17 medical officers actively providing EmOC 2 years later* • Process^4^ • *2/15 facilities providing EmOC, c-sections by medical officers* • 134 cesarean sections performed by 6 medical officers over two-year period after training	Retrospective mixed methods
Teklehaimanot et al^35^	2013	Ethiopia	Normal delivery, Instrumental delivery, Caesarean section	Government-led health system reformation and reorganization on the national level over 5 years Categories: (A) (B) (C) (D) (E) (F)	Input^1,2^ • 30 000 health extension workers trained • 3300 Primary Healthcare Units established • Process^3^ • Increased health service coverage from 64% to 92.1% • Skilled birth attendance increased from 9.5% to 16.6% • Outcome^6^ • MMR in national population decreased 33% (871 to 676/100 000)	Retrospective cohort, • pre- and post-data
Nyamtema et al^36^	2016	Tanzania	Normal delivery, Instrumental delivery, Caesarean section	Internationally funded project to upgrade 10 rural health centers to provide comprehensive obstetric services over 3-year period Categories: (A) (B)	Input^1^ • 23 medical officers trained in c-section, 44 midwives and clinical officers trained in anesthesia • Process^3,5^ • 128% of all population births occurred in these facilities (women coming from outside locations led to >100%) • 2890 c-sections performed in health centers (9% of all deliveries) • *Referrals to outside hospitals decreased by 67% (from 9% to 3%) • Outcome ^6^ • *Lower health center institutional MMR than district hospitals • *Increased MMR overall in population from 32/10^5 before to 83/10^5 after intervention*	Retrospective, • pre- and post-data
Serbanescu et al^37^	2017	Uganda, Zambia	Normal delivery, Instrumental delivery, Caesarean section	District health system strengthening (multi-partner including national government and international partners) over one year Categories: (A) (B) (C) (D) (E) (F)	Input^1,2^ • Increase in number of health providers trained in EmOC • Uganda: 316 providers; Zambia: 199 providers • Increase in number of EmOC facilities • Uganda: 7 to 16 centers, 10 to 25% of facilities; Zambia: 4 to 5 centers, 7 to 11% of facilities Process^3,4,5^ • Increase in delivery rate at EmOC facilities • Uganda: 62%; Zambia: 35% • Increase in met need for emergency obstetric care • Uganda: 46 to 66%; Zambia: 34 to 45% • Increase in c-section rates • Uganda: 23%; Zambia: 15% • Increase in complications delivered in EmOC facilities • Uganda: 25%; Zambia: 23% • Outcome ^6^ • Decreased MMR and CFR • Uganda: • MMR in regional populations decreased 30% (452 to 316/100 000) • *MMR in health facilities decreased 35% (534 to 345/100 000, * P * < .01) • *CFR in health facilities decreased 25% (2.6% to 2%, * P * < .01) • Zambia: • *MMR in health facilities decreased 35% (310 to 202/100 000, * P * < .05) • *CFR in health facilities decreased 34% (3.1% to 2%, * P * < .05)	Retrospective cohort, • pre- and post-data
Henry et al^38^	2017	Zambia	Normal delivery, Instrumental delivery, caesarean section	Multi-partner initiative to improve emergency obstetric capacity and utilization in one district including 90 health facilities, 5-year program, study measures year one results Categories: (A) (B) (D) (E)	Process^3^ • *45% increase in facility-based births (54.8% to 64.6%)	Quasi-experimental, • retrospective • pre- and post-data
**General Surgery**						
Lissouba et al^39^	2010	South Africa	Male circumcision	Community-based intervention in high-HIV-prevalence community with low circumcision rates: free services provided, active recruitment of patients over 5-month period • Categories: (A) (B) (D) (E)	Process^3,4^ • 39% (14 011) of total uncircumcised men in population underwent surgery over 12-month period at community facility • Outcome ^8^ • 92% of patients satisfied with experience	Descriptive longitudinal
Mwandi et al^40^	2011	Kenya	Male circumcision	Government-led initiative to scale-up circumcision services in high HIV burden location with low circumcision rates over 3-year period Categories: (A) (C) (D) (E) (F)	Input^1^ • 700 providers trained • Process^3,4^ • 290 000 men circumcised (increased proportion from 55% to 84% of eligible men in province) • Outcome ^7^ • Adverse events at or below 3%	Descriptive longitudinal, • pre- and post-data
Mahler et al^41^	2015	Tanzania	Male circumcision	Government-initiative to set up mobile, temporary voluntary medical male circumcision outreach services to underserved rural communities in two regions over six years Categories: (A) (B) (D)	Process^3^ • Increase from 48% of circumcisions performed in rural areas to 93% at end • 29% prevalence of male circumcision to 82% of adult male population circumcised • 267 917 total men circumcised	Descriptive longitudinal, • pre- and post-data
Amuri et al^42^	2016	Tanzania	Male Circumcision	Government-led project to offer early infant male circumcision in 8 health facilities in one region over 21 months Categories: (A)	Process^3^ • 2000 male infants circumcised (16.4% of eligible infants born at identified facilities)	Cross-sectional study
Galukande et al^43^	2016	Uganda	MC: hernia repair, lump excision, hydrocelectomy, thyroidectomy	Surgical camp to rural areas by in-country professional team over four days Categories: (D)	NA • 551 procedures performed on 536 patients over 8 sites	Descriptive, cross-sectional
O’Flynn et al^44^	2017	Burundi, Ethiopia, Kenya, Malawi, Mozambique, Rwanda, Tanzania, Uganda, Zambia, Zimbabwe		Regional multi-national training program for surgeons, with international partnerships; describes 5 years of program and outcomes Categories: (A)	Input^1^ • 212 surgeons trained in 10 countries in first 2 years • 360 “Master Trainers” trained	Descriptive, cross-sectional
**Injury**						
Washington et al^45^	2014	Myanmar	Resuscitation, suturing laceration, limb injury management, fasciotomy, amputation, airway management	International team working with community-based organizations to provide trauma simulation training to health workers; outcomes from 9 years presented Categories: (A) (B) (C)	Input^1^ • 395 community health workers trained • Process^3^ • 1232 major trauma patients received care from trained health workers over 9 years	Descriptive, cross-sectional
Tajsic et al^46^	2017	Cambodia	Open fracture management, external fixation	Trauma on-site training for local surgeons by international team over 6 years Categories: (A) (B) (C)	Input^1^ • 35 local surgeons trained from 16 hospitals in open fracture management • Process^3^ • Pilot study of 23 cases of open fracture successfully managed by training participants	Prospective interventional
**Congenital**						
Pirani et al^47^	2009	Uganda	Repair of club foot	International partnership with government to provide training program in Ponseti method over 6-year period Categories: (A) (B) (D) (E)	Input^1^ • 798 health professionals in 21 hospitals trained to provide club foot treatment	Descriptive cross-sectional
Evans et al^48^	2016	Bangladesh	Repair of club foot	International NGO partnership with government hospitals to establish club foot surgery and care in urban centers with rural satellite clinics over 4 years Categories: (A) (B) (D) (E)	Process^3^ • 17 500 children treated • Outcome ^8^ • 97% of queried parents satisfied who were available for follow-up • 99% of children at follow-up walking independently	Descriptive, prospective
**Visual Impairment **						
Sangameswaran et al^49^	2016	India	Cataract extraction	Mobile eye surgical unit run by in-country staff to provide cataract care to rural populations over 3-year period Categories: (A) (D) (E)	Process^4^ • 2021 patients in 21 remote locations underwent cataract surgery	Descriptive longitudinal
**Multiple**						
Sani et al^50^	2009	Niger	MC: Caesarean section, uterine rupture, hernia repair	Government-led initiative to launch surgery at the district hospital level over one year Categories: (A)	Process^4,5^ • 544 patients received operations • Decreased reduction in transfers to regional hospital from 82% to 52% • Outcome ^6^ • *Mortality rate for emergency c-section comparable to regional hospital: 6.25% at intervention sites, 5.7% at regional hospital*	Retrospective chart review, qualitative, • regional data review
Bolkan et al^51^	2017	Sierra Leone	MC: hernia repair, laparotomy, caesarian section	Task-sharing program through international partnership to expand provision of surgical care over 5 years Categories: (A)	Input^1^ • 48 trainees started, 9 graduated surgical assistant community health officers active in community • Process^4^ • Median of 173 operations annually performed by each surgical officer • Outcome ^6^ • Crude in-hospital mortality rate after c-section lower for indirectly supervised new surgical officers (0.4% or 6/1169) than for cases observed during training (1.2% or 8/688)	Prospective observational study

MC in specific procedure denotes most common procedures in studies targeting multiple procedures. NA signifies Not Applicable; specific outcomes consistent with our framework were not available from these studies. Studies with equivocal or negative outcomes have the outcome *italicized*. Outcome categories consistent with the Donabedian classification of outcomes are **bolded** for emphasis. An * next to an Outcome indicates a statistically significant result or change from the intervention. Population-level outcomes refer to regional population included in the study and do not represent national-level data unless otherwise indicated. Abbreviations: MVA, manual vacuum aspiration. NGO, non-governmental organization. VIA, visual inspection with acetic acid for cervical cancer screening. MMR signifies maternal mortality ratio. CFR signifies case fatality rate or direct maternal deaths divided by number of women admitted with obstetric complications. EmOC stands for Emergency Obstetric Care and signifies capacity to provide comprehensive emergency obstetric services including caesarean section. Caesarean section may be abbreviated as c-section. Intervention Categories: A: Workforce Training; B: Infrastructure/Equipment/Supplies; C: Information System; D: Community Outreach; E: Referral System/Transportation, F: Service Delivery Organization. Outcome Categories: Input^1^: Increased Workforce; Input^2^: Increase in Infrastructure; Process^4^: Increased Facility-Based Care; Process^4^: Increased Surgical Volume or Breadth; Process^5^: Decreased Referral Rates; Outcome^6^: Decreased Mortality; Outcome^7^: Decreased Complications; Outcome^8^: Patient Satisfaction.

Nine decentralization studies (33%) showed improvement in one or more outcome measures including mortality, complication rate, or patient satisfaction.^26,32,33,35,37,40,48,50,51^All studies showing improved outcomes included workforce training and investment in infrastructure, equipment, or supplies as major components of their interventions. Five out of these nine studies (55.5%) included 4 or more intervention categories.^32,35,37,40,48^

Emergency obstetric care had the most consistent data with improvement in outcomes. Of the 9 studies focusing on decentralization of obstetric care or cesarean sections, seven studies showed improved patient-level outcomes and six studies showed an improvement in maternal mortality (Table 3). Interventions took place at health centers, district hospitals, or both. All of these interventions included workforce training. Description of the outcome varied between population-level maternal mortality ratio, facility-level maternal mortality ratio, case fatality rate (direct maternal deaths/number of women admitted with obstetric complications), and mortality rate after cesarean section (Table 3).

**Table 3:** Summary of the 6 Interventions to Decentralize Emergency Obstetric Care With Associated Improvement in Maternal Mortality Outcomes

**Reference**	**Country**	**Summary**	**Mortality Outcome**
Mekbib et al^32^	Ethiopia	• Organization: The SMP • Aim: Increase the availability of EmOC services • Study period: 3 years (1999-2001) • Level: Health centers (2) • Interventions: • Upgraded to provide basic EmOC services • Equipment, materials and supplies provided • 3 months training for GPs, midwives, and other service providers in EmOC • Interventions to improve record keeping, blood supply, physical infrastructure, and community involvement	• CFR for direct maternal deaths decreased from 7.2% in 1999 to 4.6% in 2001
Kayongo et al^33^	Ethiopia, Rwanda, Tanzania	• Organization: CARE, FEMME project • Aim: Improve the availability and quality of emergency obstetric care services at district hospitals • Study period: 4 years (2001-2004) • Level: District Hospital • Interventions: • Upgrade of facilities (renovations, repairs) • Provision of equipment, essential supplies and drugs • Training in case management for obstetric complications • Strengthening of information systems • Implementation of internal quality review systems • Advocacy to develop national standards and guidelines	**Tanzania:** • CFR decreased from 3.9% to 1.9% • **Rwanda:** • CFR decreased from 2.0% to 0.9% • **Ethiopia:** CFR decreased from 7.8% to 5.2%
Sani et al^50^	Niger	• Organization: Government of Niger • Aim: Improve access to basic surgical services Study Period: 1 year (2006-2007) • Level: District Hospital Interventions: • Establishment of 12-month training general physicians to provide emergency and elective surgical procedures at rural district hospitals • University training of nurse anesthetists and surgical aides	• Mortality rate for emergency cesarean section comparable to regional hospital: • 6.25% at intervention sites, 5.7% at regional hospital
Teklehaimanot et al^35^	Ethiopia	• Organization: Ethiopian government, Health Extension Program for rural settings • Aim: Health system reform to increase health service coverage nationally • Study period: 5 years (2004-2011) • Level: Health centers, part of primary healthcare units • Interventions: • Upgrade of facilities to provide first basic, then comprehensive emergency obstetric care • Establishment of standards • Development of information system • Health workers recruited and trained • Provision of equipment, essential drugs, medical equipment, furniture, and other supplies	• MMR in national population decreased 33% (871 to 676/100 000)
Serbanescu et al^37^	Uganda, Zambia	• Organization: SMGL - multi-partner initiative • Aim: Increase the number and geographical distribution of quality basic and comprehensive EmOC • Study Period: 1 year (2012-2013) • Level: Health center, Hospitals (4 pilot districts) • Interventions: • Upgrading facility and equipment • Providing medical supplies (including blood) • Hiring, training and mentoring staff	**Uganda:** • MMR in regional populations decreased 30% (452 to 316/100 000) • MMR in health facilities decreased 35% (534 to 345/100 000, *P* < .01) • CFR in health facilities decreased 25% (2.6% to 2%, *P* < .01) **• Zambia:** • MMR in health facilities decreased 35% (310 to 202/100 000, *P* < .05) • CFR in health facilities decreased 34% (3.1% to 2%, *P* < .05)
Bolkan et al^51^	Sierra Leone	• Organization: Ministry of Health of Sierra Leone, Capacare • Aim: Increase the surgical workforce to provide emergency surgical and obstetric care to the rural population • Study period: 5 years (2011-2016) • Level: District Hospital • Intervention: • Three-year surgical task-sharing training program aiming to teach non-specialized medical doctors and associate clinicians basic surgical and obstetric skills	• Crude in-hospital mortality rate after cesarean section was lower for indirectly supervised new surgical officers (0.4% or 6/1169) than for cases observed during training (1.2% or 8/688) • Median rate of 1.4% in other sub-Saharan African countries

Abbreviations: SMP, The Save the Mothers Project; EmOC, emergency obstetric care; GPs, general practitioners; FEMME, Foundations to Enhance the Management of Maternal Emergencies; MMR, maternal mortality ratio; SGML, Saving Mothers, Giving Life. *Note:* CFR signifies case fatality rate or direct maternal deaths divided by number of women admitted with obstetric complications. Population-level outcomes refer to regional population included in the study and do not represent national-level data unless otherwise indicated.

Five studies were associated with equivocal or negative results following the intervention.^30,32,34,36,50^ Common challenges in these studies, especially for interventions focused solely on workforce training, included the need for continuous training to maintain surgical skills^32,50^ and investment in infrastructure necessary to support surgical activities.^30,34,36^ One study showed a non-significant increase in the maternal mortality ratio (32 to 83/100 000, F-test = 1.82, *P* = .18) in health centers after the intervention.^36^ This was suspected to be due to the increased number of obstetric complications treated at these facilities after the initiative.

### Regionalization (Centralization)

Eight of the 35 studies (23%) described an intervention aimed at regionalization of surgical procedures.^52-59^ Three of these studies (38%) focused on pediatric surgery specifically (Table 4).^54,57,59^ The majority of interventions included workforce training (63%) or community outreach (73%) to increase knowledge about the newly available services. Two studies demonstrated improvement in patient outcomes: decreased mortality (odds ratio [OR] = 2.09, *P* = .006) for trauma patients directly admitted to referral hospitals in Malawi versus indirect transfers and decreased complications (lower rate of poor visual acuity for centralized care, 8.5% vs 33.3%) from cataract extraction following an investment in training and infrastructure at a centralized eye center in Suriname (Table 4).^53,56^ No negative or equivocal outcomes were noted for the regionalization studies. 

**Table 4:** Description of Studies Aimed at Regionalizing Surgical Services

**Regionalization**						
**Reference**	**Year**	**Country**	**Specific Procedure**	**Intervention Description**	**Outcome Description**	**Study Design**
**Obstetric, Gynaecological, and Family Planning**						
Delamou et al^52^	2015	Guinea	Repair obstetric fistula	International partnership with on-site training, community awareness campaigns to implement obstetric fistula repair in general hospitals; outcomes evaluated over 6 years Categories: (A) (B) (D)	NA • 85% of patients (1748/2116) had a closed fistula at discharge • 79% without residual incontinence or leakage after surgery • 21% lost to follow up at 3 months	Retrospective cohort
** Injury**						
Boschini et al^53^	2016	Malawi		Analysis of mortality outcomes from direct or indirect transfer to regional hospital for trauma care over 4 years Categories: (F)	**Outcome** ^ 6 ^ • *4.2% mortality rate for indirect transfers compared to 1.6% mortality rate for direct transfers **• OR for in-hospital mortality of 2.09 for indirect vs direct transfers**	Retrospective cohort
**Congenital**						
Jenny et al^54^	2017	Multi-national	Repair of cleft lip and palate	International NGO partnership for capacity-building in cleft care; study evaluated 13 years of outcomes Categories: (A) (D) (E)	**Process** ^3,4^ • *Increase in surgical volume from 15 surgeries/hospital/year to 109 surgeries/hospital/year • *Increase in complexity of surgeries performed with alveolar bone graft use increasing from 1% to 3.4%	Descriptive longitudinal
**Visual Impairment**						
Eliah et al^55^	2008	Tanzania	Cataract extraction	Government and NGO collaboration to establish cataract care at regional hospitals in 2 districts over 2 years Categories: (A), (B), (D) (E)	**Process** • Increase in annual number of cataract surgeries performed by local surgeons 2-3 fold • Region 1: CSR increased from 216 to 546 • Region 2: CSR increased from 194 to 575	Descriptive longitudinal
Pawiroredjo et al^56^	2017	Suriname	Cataract extraction	Cataract surgical intervention program at capital city’s academic hospital – includes local and international surgeons; outcomes evaluated over 8 years Categories: (A) (B) (D) (E)	**Input** ^1^ • Increase in ophthalmologists per population (12 per one million in 2006 to 18 per one million in 2014) **Process**^3,4^ • Increased number of surgeries per ophthalmologist per year from 192 to 454 • Increase in total number of surgeries per year from 1150 to 4538 surgeries National CSR increased to 9103 **Outcome**^7^ • **Lower rate of post-operative poor visual acuity at center (6.8%) compared to other facilities prior to intervention (16.1%)**	Retrospective cohort, cross-sectional
**Multiple**						
Calisti et al^57^	2011	Eritrea	Pediatric surgery MC: anorectal malformations, release of urinary obstruction, orchiopexy	Mission trips by international team with on-site training at a referral hospital over a four-year period Categories: (A)	**Input** ^1^ • 1 local surgical resident trained to independently perform pediatric surgical procedures	Descriptive, cross-sectional
Wilson et al^58^	2012	Tanzania	Burr hole, shunt for hydrocephalus	International on-site neurosurgical training over a one-year period Categories: (A)	**Input** ^ 1 ^ • 2 local surgeons trained in neurosurgical care	Descriptive longitudinal
Merceron et al^59^	2015	Guatemala	Pediatric surgery	Centralized pediatric surgical hospital in capital city staffed by international visiting surgeons and local providers; hospital created in 2011, outcomes evaluated over next 4 years Categories: (B) (D) (E) (F)	**Process** ^3^ • Increase in surgical volume from 282 over 5 years to 2260 operations over 4 years after center was built **Outcome** ^8^ • **100% of surveyed patients rated care as good or excellent (6 or 7) on 7-point Likert scale**	Retrospective cohort, cross-sectional

Abbreviations: NGO, non-governmental organization; CSR, cataract surgical rate, or number of cataract surgeries per population in millions; OR, odds ratio‏. MC in specific procedure denotes most common procedures in studies targeting multiple procedures. NA signifies; specific outcomes consistent with our framework were not available from these studies. Outcome categories consistent with the Donabedian classification of outcomes are **bolded** for emphasis. An * next to an Outcome indicates a statistically significant result or change from the intervention. Population-level outcomes refer to regional population included in the study and do not represent national-level data unless otherwise indicated. Intervention Categories: A: Workforce Training; B: Infrastructure/Equipment/Supplies; C: Information System; D: Community Outreach; E: Referral System/Transportation, F: Service Delivery Organization Outcome Categories: Input^1^: Increased Workforce; Input^2^: Increase in Infrastructure; Process^3^: Increased Facility-Based Care; Process^4^: Increased Surgical Volume or Breadth; Process^5^: Decreased Referral Rates; Outcome^6^: Decreased Mortality; Outcome^7^: Decreased Complications; Outcome^8^: Patient Satisfaction

## Discussion

The appropriate distribution of surgical services in a health system is an essential consideration when addressing the large burden of surgical disease in LMICs. The majority of evidence in this review points to successful decentralization for high volume, low resource, and low complexity procedures such as obstetric care, cryotherapy, and male circumcision. Initiatives aimed at decentralization of emergency obstetric care were most commonly associated with improvements in mortality, however there were no studies describing regionalization of obstetric care for comparison. Regionalization was utilized more for low acuity, low volume, and highly complex conditions such as obstetric fistula repair and cleft lip and palate repair. In planning for distribution of surgical procedures and services specifically in LMICS, the domains of (1) acuity, (2) surgical volume, and (3) complexity should be addressed.

Acuity of the surgical condition is the first factor we consider in this framework. The Lancet Commission on Global Surgery proposed a goal of emergency surgical access within 2 hours, which is especially relevant for the three Bellwether procedures: cesarean section, laparotomy, and open fracture management.^60^ These procedures are designated Bellwethers, as they are markers for predicting minimum surgical capacity. It logically follows that an effort should be made to ensure these procedures are provided in district hospitals or health centers for greatest access.

Trauma surgery may be considered the surgical field with the highest acuity. There is strong evidence from HICs showing improved outcomes - specifically decreased mortality - with regionalized trauma care in the United States and elsewhere.^14,61^ The translatability of trauma care centralization from HICs to LMICs depends on the strength of the entire emergency system: robust referral systems, transportation mechanisms, and effective communication and information systems, which are often lacking.^62^ This point is well illustrated by the study in Malawi, which showed decreased mortality for trauma patients directly admitted to a tertiary hospital specializing in trauma care, as opposed to those patients who arrived as transfers from other facilities.^63^

Volume of the surgery, or prevalence of the condition requiring surgery, is the second factor which should guide surgical service distribution. In order to address the high global burden of disease attributable to surgery, decentralized facilities which are closest to the majority of the population must address the most common surgical conditions. This is consistent with prior recommendations from the WHO, advocating for the district hospital to provide immediate treatment for the “95%-99% of major life-threatening conditions amenable to surgery.”^64^ Similarly, the DCP3 includes high volume procedures such as hernia repair and cesarean section in surgical packages at the district hospital level.^3^ Emergency obstetric care in particular, with cesarean section being one of the most common surgeries worldwide, must be geographically accessible for women in order to reduce maternal and perinatal mortality.^63,65^ This review, consistent with evidence from HICs,^66,67^ points to the ability to preserve patient outcomes with decentralization of basic obstetric procedures.Of note, no studies describing the regionalization of emergency obstetric care were included in this review for comparison. The included studies show improved maternal outcomes as compared to the status quo prior to these interventions, but we are unable to conclude decentralization is superior to regionalization given this lack of evidence. More long-term data is needed to prove the sustainability of these interventions and to directly compare strategies to regionalize obstetric care.

Regionalization is recommended for low volume procedures, especially for highly complex surgeries requiring more experienced surgical staff and a large specialist multidisciplinary team. There are several studies from HICs linking higher surgical volumes with improved patient outcomes, especially for cancer surgery such as pancreatic, liver, colorectal and breast cancer surgery.^68-73^ Policies of minimum volume standards for hospitals have been applied in some HIC settings to preserve quality of care and improve patient outcomes for specific procedures.^74^ However, these policies must be considered within the context of low-resource settings, where issues such as increased travel distances to a surgical center, increased distance from patient support systems, and the potential for worsening disparities between patients treated in high- versus low-volume centers, particularly for rural populations and patients with low socioeconomic status, are particularly pertinent.^16,17,74^

The third factor considered in this framework for the distribution of surgical care is the complexity of the procedure: the demands on the technical skill and resources required to perform that procedure. Procedures where high technical expertise is required, cleft lip or palate repair for example, may be better concentrated in centralized facilities to match the distribution of specialized surgical providers and their associated multidisciplinary teams and to optimize patient volume needed to maintain standards of care.^54,75^ Regionalization is often argued to be the most cost-effective approach for these procedures due to economies of scale, but the financial burden on patients (transportation and out-of-pocket costs) should be carefully considered.^75,76^ Community outreach was an essential component of the majority of regionalization interventions in this study, emphasizing the importance of community engagement.^52,54-56^ Mobile surgical camps or intermittent travel to lower-level hospitals may be implemented to improve accessibility to these highly complex services.^66^

The strengths of this study include the focus on evidence from LMICs. This is also the first study to focus on distribution of surgical care in these settings. Extraction of surgical procedures and interventions, as well as outcomes, allowed us to determine which aspects of a study could be associated with positive outcomes. For instance, workforce training and involvement of multiple intervention categories (4 or more) were associated with improved outcomes with decentralization. This suggests that human resources are a key component of increasing access to surgical care in underserved locations, but material and system-level supports are required to be effective.

Our review is not without limitations. We used a single database (PubMed) and limited results to English, which may have contributed to selection bias. Limitations of our results include that most interventions describe financial and logistical support from HICs, and thus may not be as relevant to nationally-driven healthcare and surgical plans without initial foreign investment. Furthermore, it has been shown that public investment in universal health coverage, including access to essential surgical services, is imperative for sustainable progress towards this aim.^77^ Many outcomes are at the facility-level and not the population-level, making it difficult to generalize the results to a national-level. Most procedures were limited to decentralization or regionalization interventions and not both, making comparison between these two strategies for a particular surgery difficult. There was an overall dearth of patient-level outcomes in many of the included studies. Finally, the studies themselves were varied in terms of intervention, design, outcome choice, and quality, making it difficult to draw generalizable conclusions.

While this study provided a broad overview of the literature examining the optimal distribution of surgical services, this question would be better answered through large-scale population-level research. Ideal studies to fit this aim would directly compare decentralization and regionalization of specific procedures, cost-effectiveness of each approach, and the result on patient outcomes. Given the challenges and feasibility of conducting these large-scale interventions, modeling studies may be more practical to answer this question.

## Conclusion

This review of evidence around decentralization and regionalization of surgical services in LMICs has revealed mortality benefits for interventions aimed at decentralizing obstetric care to the district hospital and health center levels. While more evidence is needed to provide robust recommendations for the optimal distribution of procedures in LMICs, there are several domains which should be considered in each specific context: the acuity of the condition, the surgical volume of the procedure, and the complexity of the operation. Factors affecting patient access to surgical care, such as referral and transportation networks, must be integrated within this framework.

## Acknowledgements

We thank The Lancet Global Health Commission on High Quality Health Systems in the SDG Era, its chair, Dr. Margaret Kruk, and one of its researchers, Dr. Sanam Roder-DeWan (both of whom are affiliated with Harvard T.H. Chan School of Public Health, Boston, MA, USA) for their assistance with developing the concept of the study and reviewing the manuscript.

## Ethical issues

Not applicable.

## Competing interests

The concept of this study was formed through collaboration with the research team involved in The Lancet Global Health Commission on High Quality Health Systems in the SDG Era.

## Authors’ contributions

KRI, ES, KS, EJB, IC, and KAV contributed to the conception and design of the study. ES and BLP performed the data acquisition and article selection. KRI, ES, BLP, KS, EJB, IC, and KAV contributed to analysis and interpretation of the data. KRI and ES performed the analysis of results. KRI and ES drafted the manuscript. JGM and MGS supervised all phases of the study and manuscript preparation. All authors contributed to critical revisions and have seen and approved the final manuscript.

## Authors’ affiliations

^1^Program in Global Surgery and Social Change, Harvard Medical School, Boston, MA, USA. ^2^General Surgery Department, University of California Davis Medical Center, Sacramento, CA, USA. ^3^Lund University, Lund, Sweden. ^4^Brigham and Women’s Hospital, Boston, MA, USA. ^5^Icahn School of Medicine at Mount Sinai, New York City, NY, USA. ^6^University of Pennsylvania, Philadelphia, PA, USA. ^7^Stanford University School of Medicine, Stanford, CA, USA. ^8^Department of Plastic and Oral Surgery, Boston Children’s Hospital, Boston, MA, USA. ^9^Massachusetts Eye and Ear Infirmary, Boston, MA, USA.

## Supplementary files


Supplementary file 1 contains all search terms.
