# Supplementary material for: Decentralization and Regionalization of Surgical Care: A Review of Evidence for the Optimal Distribution of Surgical Services in Low- and Middle-Income Countries
Source: Int J Health Policy Manag. 2019 Jun 17;8(9):521–37. doi: 10.15171/ijhpm.2019.43 (PMC6815989; doi:10.15171/ijhpm.2019.43)
Supplement: Supplementary file 1 — contains all search terms. [file ijhpm-8-521-s001.pdf]

Supplementary file 1

LMIC search string

"Developing Countries"[mesh] OR developing countr*[tiab] OR developing nation*[tiab] OR less developed countr*[tiab] OR less developed nation*[tiab] OR third world nation*[tiab] OR third world countr*[tiab] OR under developed nation*[tiab] OR underdeveloped nation*[tiab] OR under developed countr*[tiab] OR underdeveloped nation*[tiab] OR middle income countr*[tiab] OR middle income nation*[tiab] OR low income countr*[tiab] OR low income nation*[tiab] OR poor countr*[tiab] OR poor nation*[tiab] OR lmic[tiab] OR lmics[tiab] OR "Africa"[mesh] OR "Asia"[mesh] OR "South America"[mesh] OR "Latin America"[mesh] OR "Central America"[mesh] OR africa[tiab] OR asia[tiab] OR south america[tiab] OR latin america[tiab] OR central america[tiab] OR Afghanistan*[tiab] OR Albania*[tiab] OR Algeria*[tiab] OR Samoa*[tiab] OR Angola*[tiab] OR Armenia*[tiab] OR Azerbaijan*[tiab] OR Bangladesh*[tiab] OR Bengali[tiab] OR Belarus*[tiab] OR Belize[tiab] OR Benin[tiab] OR Bhutan*[tiab] OR Bolivia*[tiab] OR Bosnia*[tiab] OR Herzegovina*[tiab] OR Botswana*[tiab] OR Brazil*[tiab] OR Bulgaria*[tiab] OR Burkina Faso[tiab] OR Burundi*[tiab] OR Cabo Verd*[tiab] OR Cape Verd*[tiab] OR Cambodia*[tiab] OR Cameroon*[tiab] OR Central African*[tiab] OR Chad*[tiab] OR China[tiab] OR Chinese[tiab] OR Colombia*[tiab] OR Comoros[tiab] OR Congo[tiab] OR Costa Rica*[tiab] OR Cote d'Ivoire[tiab] OR Ivory Coast[tiab] OR Cuba[tiab] OR Cuban[tiab] OR Djibouti[tiab] OR Dominica* [tiab] OR Ecuador[tiab] OR Egypt[tiab] OR El Salvador*[tiab] OR Eritrea*[tiab] OR Ethiopia*[tiab] OR Fiji*[tiab] OR Gabon*[tiab] OR Gambia*[tiab] OR Georgia*[tiab] OR Ghana*[tiab] OR Grenada*[tiab] OR Guatemala*[tiab] OR Guinea*[tiab] OR Guyan*[tiab] OR Haiti*[tiab] OR Hondura*[tiab] OR India[tiab] OR Indian*[tiab] OR Indonesia*[tiab] OR Iran*[tiab] OR Iraq*[tiab] OR Jamaica*[tiab] OR Jordan*[tiab] OR Kazakh*[tiab] OR Kenya*[tiab] OR Kiribati[tiab] OR People's Republic of Korea[tiab] OR North Korea[tiab] OR Kosovo[tiab] OR Kosovar* [tiab] OR Kyrgyz*[tiab] OR Lao[tiab] OR Laos[tiab] OR Laotian*[tiab] OR Lebanon[tiab] OR Lebanes*[tiab] OR Lesotho[tiab] OR Liberia*[tiab] OR Libya*[tiab] OR Macedonia*[tiab] OR Madagascar*[tiab] OR Malawi*[tiab] OR Malaysia*[tiab] OR Maldives[tiab] OR Mali[tiab] OR Marshall Island*[tiab] OR Mauritania*[tiab] OR Mauriti*[tiab] OR Mexico[tiab] OR Mexican*[tiab] OR Micronesia*[tiab] OR Moldova*[tiab] OR Mongolia*[tiab] OR Montenegr*[tiab] OR Morocc*[tiab] OR Mozambique[tiab] OR Myanmar[tiab] OR Burmese*[tiab] OR Burma[tiab] OR Namibia*[tiab] OR Nepal*[tiab] OR Nicaragua*[tiab] OR Niger*[tiab] OR Pakistan*[tiab] OR Palau[tiab] OR Panama*[tiab] OR Paraguay*[tiab] OR Peru*[tiab] OR Philippin*[tiab] OR Romania*[tiab] OR Rwanda*[tiab] OR Samoa*[tiab] OR Sao Tome[tiab] OR Principe[tiab] OR Senegal*[tiab] OR Serbia*[tiab] OR Sierra Leone*[tiab] OR Solomon Island*[tiab] OR Somalia* [tiab] OR South Africa*[tiab] OR Sri Lanka[tiab] OR St Lucia[tiab] OR Saint Lucia[tiab] OR St Vincent[tiab] OR Saint Vincent[tiab] OR Grenadines[tiab] OR Sudan*[tiab] OR Suriname*[tiab] OR Swaziland*[tiab] OR Syria*[tiab] OR

Tajik*[tiab] OR Tanzania*[tiab] OR Thai*[tiab] OR Timor*[tiab] OR Togo*[tiab] OR Tonga*[tiab] OR Tunisia*[tiab] OR Turkey[tiab] OR Turkish[tiab] OR Turkmen*[tiab] OR Tuvalu*[tiab] OR Uganda*[tiab] OR Ukrain*[tiab] OR Uzbeki*[tiab] OR Vanuatu*[tiab] OR Vietnam*[tiab] OR Viet nam*[tiab] OR West Bank[tiab] OR Gaza*[tiab] OR Palestin*[tiab] OR Yemen*[tiab] OR Zambia*[tiab] OR Zimbabw*[tiab]

Decentralization/Regionalization Search String

(district hospital) OR (community health center) OR (primary health centre) OR (primary health center) OR (community facility) OR rural OR decentralization OR regionalization OR (First-level hospital) OR (referral hospital) OR (specialised hospital) OR (specialized hospital) OR (devolution) OR (devolved) OR (devolve) OR (devolvement) OR (devolving)

Procedure Search String

(cesarean birth) OR (cesarean section) OR (vasectomy) OR (tubal ligation) OR (ectopic pregnancy) OR (hysterectomy) OR (post-partum hemorrhage) OR (bleeding control) OR (uterine rupture) OR (manual vacuum aspiration) OR (vacuum extraction) OR (cervical dilation) OR (curettage) OR (circumcision) OR (bowel perforation) OR (appendectomy) OR (appendicitis) OR (bowel obstruction) OR (colostomy) OR (cholecystectomy) OR (hernia) OR (hernia repair) OR (hydrocelectomy) OR (urinary obstruction) OR (suprapubic cystostomy) OR (thoracostomy) OR (trauma laparotomy) OR (fracture reduction) OR (open fracture) OR (debridement) OR (external fixator) OR (fracture traction) OR (traction) OR (escharotomy) OR (fasciotomy) OR (traumatic amputation) OR (femur amputation) OR (under-knee amputation) OR (skin grafting) OR (burr hole) OR (hydrocephalus) OR (cataract repair) OR (obstetric fistula) OR (cleft lip) OR (club foot) OR (anorectal malformation) OR (Hydrocelectomy) OR (Hirschsprung’s disease) OR (abscess drainage) OR (septic arthritis) OR (osteomyelitis) OR (cryotherapy) OR (resuscitation) OR (surgical airway) OR (tracheostomy)
